# Observation of correlated X-ray scattering at atomic resolution

**DOI:** 10.1098/rstb.2013.0315

**Published:** 2014-07-17

**Authors:** Derek Mendez, Thomas J. Lane, Jongmin Sung, Jonas Sellberg, Clément Levard, Herschel Watkins, Aina E. Cohen, Michael Soltis, Shirley Sutton, James Spudich, Vijay Pande, Daniel Ratner, Sebastian Doniach

**Affiliations:** 1 Department of Applied Physics, Menlo Park, CA 94025, USA; 2 Department of Chemistry, Menlo Park, CA 94025, USA; 3 Department of Biochemistry, Stanford University School of Medicine, Menlo Park, CA 94025, USA; 4 SLAC National Accelerator Laboratory, Menlo Park, CA 94025, USA; 5 Department of Physics, AlbaNova University Center, Stockholm University, S-106 91 Stockholm, Sweden; 6 Department of Geological and Environmental Sciences, Stanford University, Stanford CA 94305, USA; 7 Aix-Marseille University, CNRS, IRD, CEREGE UM34, 13545 Aix-en-Provence, France

**Keywords:** X-ray angular correlations, Bragg peak correlations, silver nanoparticles, atomic resolution X-ray scattering, synchrotron radiation, solution ensemble

## Abstract

Tools to study disordered systems with local structural order, such as proteins in solution, remain limited. Such understanding is essential for e.g. rational drug design. Correlated X-ray scattering (CXS) has recently attracted new interest as a way to leverage next-generation light sources to study such disordered matter. The CXS experiment measures angular correlations of the intensity caused by the scattering of X-rays from an ensemble of identical particles, with disordered orientation and position. Averaging over 15 496 snapshot images obtained by exposing a sample of silver nanoparticles in solution to a micro-focused synchrotron radiation beam, we report on experimental efforts to obtain CXS signal from an ensemble in three dimensions. A correlation function was measured at wide angles corresponding to atomic resolution that matches theoretical predictions. These preliminary results suggest that other CXS experiments on disordered ensembles—such as proteins in solution—may be feasible in the future.

## 1. Introduction

In a pioneering paper, Kam [1] showed that correlated X-ray scattering (CXS) from an ensemble of randomly oriented particles could in principle reveal information about the internal structure of the particles beyond usual small and wide angle solution scattering measurements. The extraction of such information in the absence of an ordered system (e.g. a crystal) can be beneficial in biological studies, as many biological systems with well-defined local structural order are inherently disordered at large length scales.

In order to gauge the feasibility of Kam's method at atomic resolution and to assess the associated difficulties, we conducted experiments measuring CXS from silver nanoparticle (NP) solutions at wide angles. Crucially, each measurement was conducted on an ensemble of NPs oriented randomly in three dimensions, extending previous experimental work done in two dimensions [2], at small angles [3,4] or on single particles [5,6].

From these experiments, we obtained empirical correlation functions between all pixel pairs in two silver Bragg rings, corresponding to Miller indices 111 and 200. Preliminary analysis of the three correlation functions (two rings correlated with themselves and with each other) show sharp peaks consistent with analytical and simulated predictions based on the crystal structure of silver.

By successfully measuring CXS signal from randomly oriented ensembles of silver NPs, we have demonstrated the effectiveness of Kam's method at atomic resolution. This experiment will serve as a benchmark for future experiments involving ensemble exposures. Refinement of our experimental technique on a well-characterized sample (i.e. silver NPs) will also help facilitate the extension of CXS for studies of weaker scatterers in solution.

## 2. Theory

We briefly review the portions of the earlier study [1] relevant to this paper. Let *S*(***q***, *ω*) represent the structure factor of an isolated particle in solution, i.e.2.1
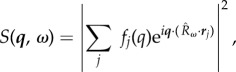
where ***q*** is the scattering momentum transfer vector, ***r****_j_* and *f_j_*(*q*) are the coordinates and form factor of the *j*th atom in the particle, respectively, *ω* is a triple of Euler angles, 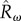 is a three-dimensional rotation operator and the sum is over all atoms in the particle.

Kam showed that if the distribution of particle rotations dictated by 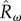 is isotropic, distributed uniformly over the rotation group SO(3), then the structure factor correlation function,2.2
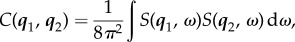
may be extracted from a CXS measurement where one repeatedly records snapshots of an *N*-particle solution, with each snapshot representing a unique ensemble of the particles frozen in three-dimensional space. Kam showed (assuming negligible inter-particle scattering interference) that the empirical correlation function averaged over shots would converge to (2.2), i.e.2.3
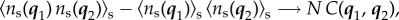
where *n*_s_(***q***) is the total photons scattered from all particles in snapshot *s* into a pixel along scattering vector ***q***, and *N* is the number of exposed particles in each shot (assumed here to be constant). Neglecting inter-particle scattering interference and assuming a large number of incident photons per shot,2.4
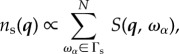
where *Γ*_s_ represents the set of particle orientations for shot *s*. Note that the correlation function on the left-hand side of (2.3) contains the isotropically averaged scattering, which is subtracted in order to recover (2.2).

In this special case of an isotropically oriented sample, Kam proved that the absolute orientation of the ***q***_1_, ***q***_2_ scattering pair in the correlation function (2.2) is irrelevant; the relevant covariates are only the magnitudes *q*_1_, *q*_2_ and the angle between the vectors, *ψ*. Thus, the correlation function can be written as
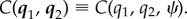


Experimentally, we statistically estimate this function by taking angular correlations in the detector plane; let *ϕ* be the azimuthal coordinate of ***q*** projected into the plane perpendicular to an incident beam (corresponding to the azimuthal coordinate of a pixel measuring ***q*** on a far-field planar detector). Let Δ = *|ϕ*_1_ − *ϕ*_2_*|* be the angle between two such vector projections. Then define the average angular correlation of ring intensity fluctuations as2.5
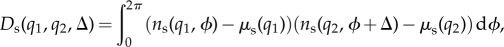
and its average,2.6
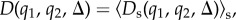
where *μ*_s_(*q*) is the average photon counts at *q* in snapshot *s* (by subtracting the average from each shot before correlating, we are effectively performing the subtraction on the LHS of equation (2.3)). Assuming randomly oriented particles, negligible inter-particle scattering (wide angles and/or a dilute sample) and a sufficiently large number of snapshots, *D*(*q*_1_, *q*_2_, Δ) will converge to a statistical estimate of Kam's correlation *C*(*q*_1_, *q*_2_, *ψ*), up to a trigonometric conversion of Δ (the angle between ***q***_1_, ***q***_2_ projections on the plane of the detector) and *ψ* (the angle between ***q***_1_, ***q***_2_). This is given by the relationship2.7
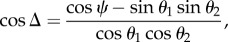
where 2*θ*_1_ and 2*θ*_2_ are the standard scattering angles of ***q***_1_ and ***q***_2_, respectively (by standard scattering angle, we mean the angle between the forward beam direction and the scattered X-rays).

**Figure 1.**
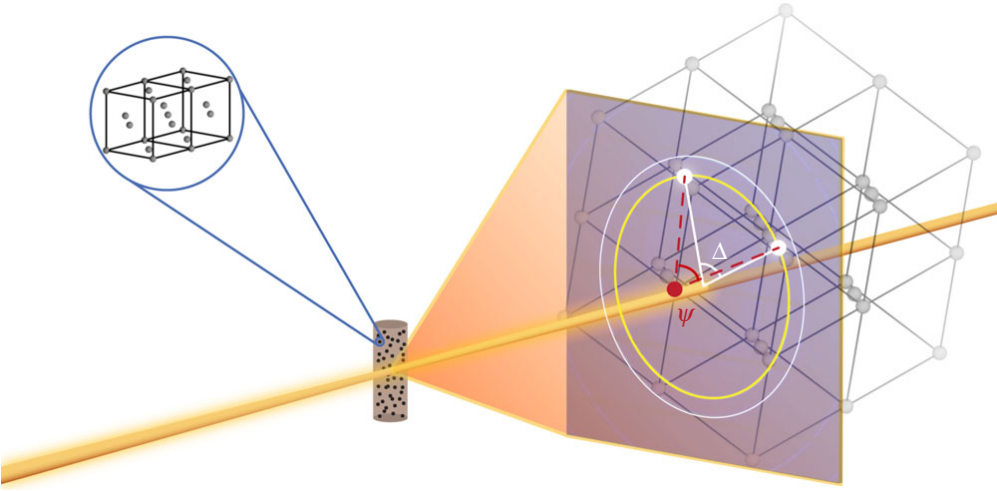
Experimental set-up: a kapton capillary filled with a solution of silver NPs (face-centred-cubic). Bragg rings *q*_111_ and *q*_200_ are illustrated by circles on the detector plane. At least one of the exposed NPs happens to be oriented such that two reciprocal lattice (body-centred-cubic) peaks are intersecting the detector at *q*_111_. Dashed lines represent the scattering vectors (separated by the angle *ψ*), and solid lines represent the projection of those vectors onto the detector plane (separated by the angle Δ). Artwork courtesy of Gregory M. Stewart (SLAC). (Online version in colour.)

We have computed the expected angular correlation function (2.6) for the silver NPs studied as a benchmark for our experimental results (figure 3*b*). A silver NP may be represented by a simple model consisting of a face-centred-cubic lattice cut-off by a spherical boundary. The scattering can then be represented by reciprocal lattice vectors intersecting the Ewald sphere and giving rise to Bragg peaks. Hence, each snapshot records a series of Bragg rings (henceforth, we will only consider scattering vectors which meet the Bragg condition for silver, denoted by a set of Miller indices, ***q****_hkl_*). The sub-population of all NP orientations that simultaneously subtend two Bragg peaks on the Ewald sphere give rise to angular correlations. Therefore, CXS signal appears at values of Δ corresponding to the geometry of the reciprocal lattice and the angles between reciprocal lattice vectors (figure 1). For instance, *D*(*q*_111_, *q*_111_, Δ) should display two peaks due to the two reciprocal lattice vectors of magnitude *q*_111_ whose angles of separation *ψ*_1_ and *ψ*_2_ are given by cos*ψ*_1_ = 1/3 and cos*ψ*_2_ = −1/3, respectively. The peak locations are found using (2.7): Δ_1_ = arccos[−2*/*3 cos^2^*θ*_111_ + 1] and Δ_2_ = arccos[−4*/*3cos^2^*θ*_111_ + 1], where 2*θ*_111_ is the standard scattering angle of Bragg vector ***q***_111_.

It is clear that the function *C*(***q***_1_, ***q***_2_) in (2.2) contains information about the structure of the particles under study. Even though one can show that inverting a complete set of such correlations to an image of the electron density (at arbitrary resolution) is an underdetermined problem [7], we would like to emphasize that (2.2) can be calculated using a model for the structure factor (2.1) defined by a particle's atomic positions. This opens up the possibility for refining such a model against a CXS dataset using (2.3) [8–10], especially in cases where prior information (such as protein primary sequence) can be included. Other routes to analysing structure given CXS data include iterative phasing [11], intentional alignment of particles [12] or direct analysis of local internal symmetries of the system [13,14].

Theoretical results suggest the CXS signal to noise scales as the square-root of the number of recorded shots, but is independent of the number of illuminated particles *N*, for large *N* [1,3,15]. These facts were employed to optimize our experimental design, which emphasized collecting a large number of independent snapshots.

## 3. Material and methods

Data from 15 496 X-ray diffraction images [16] of silver NP solution were collected and analysed at the micro-focus crystallography beamline (12–2) at SSRL. We prepared a sample containing an estimated 10^9^ 20 nm NPs per snapshot in the illuminated volume, but we observed significant numbers of NPs that were larger. Samples were loaded and oriented in the X-ray beam using the Stanford automated mounting system [17], controllable from the experimental hutch. Using a liquid nitrogen-cooled double crystal monochromator, we tuned the beam energy to 17 keV. The beam was focused down to about 20 × 50 μm^2^ using Rh-coated Kirkpatrick-Baez mirrors and had a flux of 2 × 10^12^ photons per second. Snapshots were recorded on a Dectris Pilatus 6 M photon counting detector.

To successfully measure a correlation function via the scheme (2.3), the sample must be frozen in time and space. Any random motion owing to diffusion of particles will reduce the scattering correlation, which is a function of the particle structure and orientation (2.1). In attempts to prevent diffusion during the long exposure times (order 1 s) necessary to scatter a sufficient number of photons to measure a correlation signal at a synchrotron, we cooled the sample using an Oxford Instruments Cryojet, promoting particle immobilization during each exposure. By repeatedly exposing the same sample location, we estimated that the average NP will rotate by one Scherrer width in about 6 s of constant exposure. This is about a factor of 10 longer than the average exposure time during the actual experiment (0.7 s). Further, over the course of a 12 s exposure to the same sample location, the *q*_111_ Bragg ring intensity decreased by 1.2%. Thus, at an exposure time of 0.7 s, our samples experienced insignificant photo-damage.

The silver NPs, coated in PVP, were synthesized following a protocol described elsewhere [18]. In order to prevent the formation of solvent crystals at the low temperature, the NPs were concentrated and suspended in 80% glycerol and 3% agarose with a final concentration of 350 mg ml^−1^. The solutions were held in kapton capillaries (500 and 600 µm inner and outer diameter, respectively) and flash frozen in liquid nitrogen. Kapton and glycerol scatter into relatively lower angles and did not corrupt our silver NP signal.

Our goal was to record as many snapshots as possible, each one representing a different ensemble of particle orientations frozen in time. The sample holder was equipped to automatically rotate the capillary 150° about its longitudinal axis, perpendicular to the beam. Photon counts were read out and reset every 0.7 s as the capillary rotated 0.3° under continuous beam irradiation, yielding 500 shots per 150° rotational scan. After 0.3° of capillary rotation, approximately 50 ± 5% of the *q*_111_ Bragg peaks no longer intersected the Ewald sphere; this rotation setting lowered the effective number of snapshots in our experiment yet increased the amount of information at our disposal. Every 500 shots, between scans, the capillary was moved longitudinally so as to always probe different regions of the sample.

**Figure 2.**
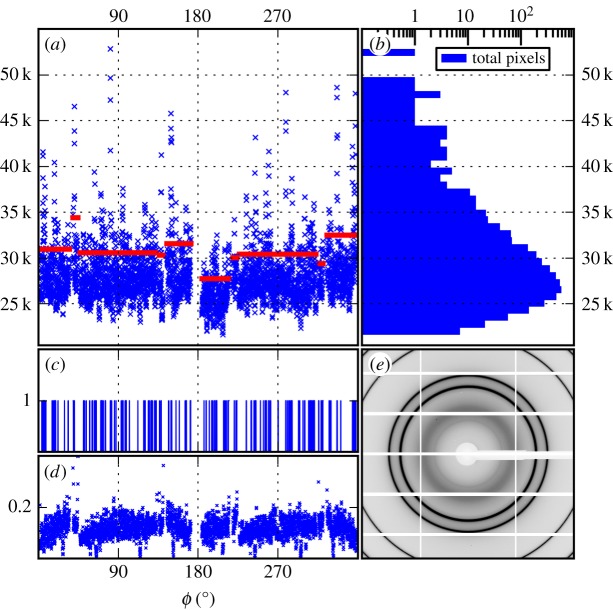
(*a*) Representative measurement of one X-ray snapshot, *n*_s_(*q*_111_, *ϕ*), i.e. the photon counts around the Bragg ring at *q*_111_. The horizontal bars show the binary cut-off along the ring for each module. Here, the *q*_111_ ring spans 10 modules in total. (*b*) Histogram of the intensities in (*a*) on a log scale of photon numbers. Note the tail at higher intensities, indicating the presence of large particles. (*c*) Result of applying the binary filter to (*a*). On average, a binary shot at *q*_111_ is 10% ones, 77% zeros and 13% masked pixels (masked pixels are ignored in the analysis). (*d*) The average binary intensity from a scan of 500 snapshots. Note systematic structure in the intensity response, indicating intra-module pixel variations. (*e*) Typical snapshot showing the Bragg rings at *q*_111_ and *q*_200_. Low/high intensities shown in white/black. (Online version in colour.)

A bicubic interpolation algorithm was used to convert the Cartesian pixel lattice to polar coordinates for calculation of (2.5). Using the Scherrer equation [8] relating the width of a Bragg ring to the average NP size, we concluded that the majority of silver NPs in each snapshot were roughly 20 nm in diameter. Histograms of photon counts into the *q*_111_ Bragg ring indicate a rather large distribution of particle sizes (figure 2*b*) which we discuss in §4.

## 4. Results and discussion

Here, we report on our attempts to resolve (2.6) at the two brightest silver Bragg rings. Specifically, we calculated *D*(*q*_111_, *q*_200_, Δ), *D*(*q*_111_, *q*_111_, Δ) and *D*(*q*_200_, *q*_200_, Δ).

Convergence of the CXS signal is complicated by the inherent statistical noises described in [1,7], and CXS measurement is sensitive to systematic artefacts associated with the experimental conditions [3], e.g. detector artefacts. The Pilatus 6 M detector is made up of 60 modules separated by 1–2 mm gaps, and each Bragg ring subtends multiple modules. The overall electronic response of each module is slightly different, causing systemic anisotropies which lead to detector intensity correlations that dominate the sample CXS. We recorded three sets of data corresponding to three different sample-detector distances, and each set had unique detector artefacts.

**Figure 3.**
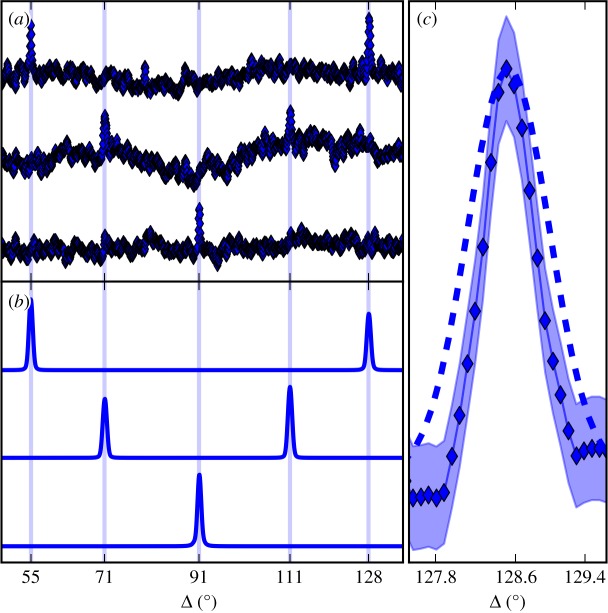
(*a*) From top to bottom, measured correlation functions *D*(*q*_111_, *q*_200_, Δ), *D*(*q*_111_, *q*_111_, Δ) and *D*(*q*_200_, *q*_200_, Δ) from 20 nm silver NPs. We truncated the angular range to highlight the correlation peaks. Regions not shown contain artefacts similar to those on the figure, with nothing greater in magnitude than the CXS peaks. (*b*) Corresponding simulations of the correlations plotted in (*a*). Vertical lines mark analytical predictions. (*c*) Simulation (dashed line) and measurement (diamond marker) of a correlation peak width. The simulation was for 20 nm particles. Peak width scales inversely with particle size, hence we expect the measured CXS resulted from particles larger than 20 nm. Shading represents 95% confidence intervals (±1.96 × s.e.) on the measurement. (Online version in colour.)

**Figure 4.**
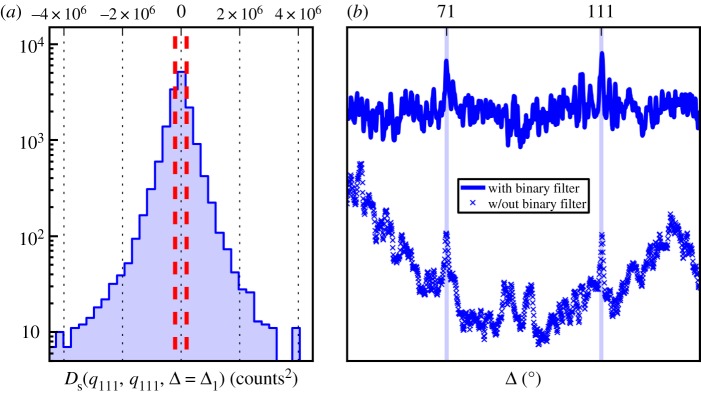
Shows the shot-to-shot variance in the correlation function (2.5). Large variance is due to the presence of large particles. We illustrate the effectiveness of the binary filter at removing systematic error induced correlations, even when the large particle fluctuations are minimized. (*a*) Histogram of *D*_s_(*q*_111_, *q*_111_, Δ = Δ_1_) over all shots on a log scale (raw data without any filter). Outliers were observed with values up to order 1 × 10^8^ and were removed (not shown). The large tails indicate the presence of large crystals in the data which can contribute to correlations. Red dashed lines mark ±200 k counts^2^. (*b*) Plots of *D*(*q*_111_, *q*_111_, Δ) for filtered (upper) and unfiltered (lower) data. The average was taken over the 4208 shots where both of the following criterion were met: −200 k counts^2^ ≤ *D*_s_(*q*_111_, *q*_111_, Δ = Δ_1_) ≤ 200 k counts^2^; −200 k counts^2^ ≤ *D*_s_(*q*_111_, *q*_111_, Δ = Δ_2_) ≤ 200 k counts^2^. In the unfiltered plot, artificial correlations due to detector and sample anisotropies are similar in magnitude to the true correlation signal. The vertical blue lines mark the analytical prediction of CXS signal (Δ_1_ and Δ_2_). (Online version in colour.)

Unprocessed estimates of the correlation function (2.6) in the data we obtained are dominated by such systematic artefacts. To mitigate these effects, we apply a binary filter to the data (figure 2). We define the filter as follows: let {***q****_hkl_*}*_m_* represent the set of Bragg pixels on the *m*th detector module (1 ≤ *m* ≤ 60). Let *μ_m_* and *σ_m_* represent the average and standard deviation of *n*_s_(***q***) for all ***q*** ∈ {***q****_hkl_*}*_m_*, respectively. Then for each ***q*** ∈ {***q****_hkl_*}*_m_*, we apply the photon count filter
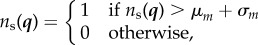
i.e. per module, all Bragg pixels less than 1 s.d. above the mean are set to zero and the rest are set to unity. The threshold *μ_m_* + *σ_m_* was sufficient for our purposes and was not optimized. The binary filter is a simple method for emphasizing the local variations from the sample compared to the background fluctuations of the detector; placing each detector module on the same scale removes correlations between modules. After applying the filter and calculating (2.6), the resulting correlations reveal peaks that match the simulations and analytical predictions (figure 3). We note that while the binary filter emphasizes larger particles, there are still many particles per shot after filtering (figure 2*c*), and the CXS signal is not affected by removing contributions from the largest particles (figure 4). Despite the filtering, variations remain within each module which bias the binary filter selection (figure 2*d*) and lead to the low-frequency background correlations shown in figure 3*a*. The binary filter is an intermediate stage in our on-going analysis; it provides a stepping stone to better data refinement techniques and CXS extraction.

As mentioned in §3, we infer from the width of the Bragg rings that the majority of our sample consists of 20 nm silver NPs (approx. 10^9^ per snapshot). From tabulated coherent atomic cross sections [19], we estimate that each 20 nm silver NP scatters roughly 1.6 photons per 0.7 s exposure. We can further estimate that roughly 8.3% of these NPs scatter into the Bragg ring at *q*_111_ on the detector. This is done by computing the volume each reciprocal lattice point occupies as the particle undergoes a complete set of rotations and then determining which fraction of that volume intersects the Ewald sphere. For this calculation, the reciprocal lattice site diameters were computed using the Scherrer equation [8]. These estimates are consistent with the average photon counts per pixel per snapshot in the *q*_111_ Bragg ring, approximately 4 × 10^4^.

However, many of the snapshots contained pixels at *q*_111_ reporting more than 10^5^ photon counts, much greater than one would expect given Poisson statistics, where fluctuations are order square-root of the mean. We therefore suspect our sample included a broad distribution of particle sizes, with larger particles scattering more photons, and thus larger particles contributing to the correlations. While the width of the powder ring indicates that the majority of NPs were roughly 20 nm, we can employ the Scherrer equation to relate the width of the correlation peak to the contributing particle size (figure 3*c*). The width of the measured correlation peak is consistent with 50 nm particles, thus we conclude that the measured correlations were most likely generated by scattering off of thousands of more than or equal to 50 nm particles per shot. We emphasize that this is an estimated lower bound on particle size; NPs more than 50 nm would have even narrower Bragg reflections. Of all 50 nm silver NP orientations, we estimate that roughly 0.19% will simultaneously subtend two Bragg peaks on the detector into *q*_111_, 0.06% will simultaneously subtend two Bragg peaks on the detector into *q*_200_, and 0.15% will simultaneously subtend one Bragg peak into each of *q*_111_, *q*_200_.

We note that convergence of the correlations was not limited by the statistical background noise owing to the large number of uncorrelated photons (figure 2*c*). At present, the main impediment to accurate measurements of CXS arises from anisotropy artefacts induced by the experimental methods, in particular the detector system. We have been able to partially overcome background correlations by nonlinear binary filtering and are continuing data processing with more sophisticated methods (figure 4).

## 5. Conclusion

Kam's original results [1] show that the correlator (2.2), which may be directly calculated from the structure factor (2.1), can be derived from measurement of an ensemble of particles. Hence, accurate measurement of CXS in the three-dimensional {*q*_1_, *q*_2_, Δ} space can lead to constraints on a sample's electron density model, providing a route to iterative refinement of the sample structure (as in [20]).

Our preliminary results reveal atomic scale information regarding the internal structure of an NP from a bulk sample containing of order 10^9^ randomly oriented particles. Where conventional powder diffraction analysis techniques observe two signal peaks (*q*_111_ and *q*_200_), CXS observes five, information that could be used for identifying space groups. These results on silver NPs suggest that it should be feasible to obtain atomic scale constraints on models of particles with approximately known structure, provided one can effectively correct for detector-anisotropy-induced correlations. With the much brighter pulses from X-ray free-electron lasers, and with advances in data refinement, it should be possible to extend the results to biomolecules in solution [21,22].

## References

[1] Kam Z 1977 Determination of macromolecular structure in solution by spatial correlation of scattering fluctuations. Macromolecules 10, 927–934. (10.1021/ma60059a009)

[2] Saldin D Poon H Bogan M Marchesini S Shapiro D Kirian R Weierstall U Spence J 2011 New light on disordered ensembles: *ab initio* structure determination of one particle from scattering fluctuations of many copies. Phys. Rev. Lett. 106, 115501 (10.1103/PhysRevLett.106.115501) 21469876

[3] Kam Z Koch MH Bordas J 1981 Fluctuation X-ray scattering from biological particles in frozen solution by using synchrotron radiation. Proc. Natl Acad. Sci. USA 78, 3559–3562. (10.1073/pnas.78.6.3559) 6943555 PMC319609

[4] Wochner P 2009 X-ray cross correlation analysis uncovers hidden local symmetries in disordered matter. Proc. Natl Acad. Sci. USA 106, 11 511–11 514. (10.1073/pnas.0905337106) PMC2703671 20716512

[5] Kam Z Gafni I 1985 Three-dimensional reconstruction of the shape of human wart virus using spatial correlations. Ultramicroscopy 17, 251–262. (10.1016/0304-3991(85)90092-0) 3003997

[6] Starodub D 2012 Single-particle structure determination by correlations of snapshot X-ray diffraction patterns. Nat. Commun. 3, 1276 (10.1038/ncomms2288) 23232406

[7] Elser V 2011 Strategies for processing diffraction data from randomly oriented particles. Ultramicroscopy 111, 788–792. (10.1016/j.ultramic.2010.10.014) 21093151

[8] Liu H Poon BK Saldin DK Spence JCH Zwart PH 2013 Three-dimensional single-particle imaging using angular correlations from X-ray laser data. Acta Crystallogr. A 69, 365–373. (10.1107/S0108767313006016) 23778093

[9] Chen G Zwart PH Li D 2013 Component particle structure in heterogeneous disordered ensembles extracted from high-throughput fluctuation X-ray scattering. Phys. Rev. Lett. 110, 195501 (10.1103/PhysRevLett.110.195501) 23705716

[10] Saldin DK Shneerson VL Fung R Ourmazd A 2009 Structure of isolated biomolecules obtained from ultrashort X-ray pulses: exploiting the symmetry of random orientations. J. Phys. Condens. Matter 21, 134014 (10.1088/0953-8984/21/13/134014) 21817489

[11] Saldin DK Poon HC Shneerson VL Howells M Chapman HN Kirian RA Schmidt KE Spence JCH 2010 Beyond small-angle X-ray scattering: exploiting angular correlations. Phys. Rev. B 81, 174105 (10.1103/PhysRevB.81.174105)

[12] Poon HC Schwander P Uddin M Saldin DK 2013 Fiber diffraction without fibers. Phys. Rev. Lett. 110, 265505 (10.1103/PhysRevLett.110.265505) 23848897

[13] Kurta RP Altarelli M Weckert E Vartanyants IA 2012 X-ray cross-correlation analysis applied to disordered two-dimensional systems. Physical Review B 85, 184204.

[14] Kurta RP Altarelli M Vartanyants IA 2013 X-ray cross-correlation analysis of disordered ensembles of particles: potentials and limitations. Advances in Condensed Matter Physics 2013, 15.

[15] Kirian RA Schmidt KE Wang X Doak RB Spence JCH 2011 Signal, noise, and resolution in correlated fluctuations from snapshot small-angle X-ray scattering*.* Phys. Rev. E 84, 011921 (10.1103/PhysRevE.84.011921) 21867227

[16] Mendez D Lane TJ Ratner D Doniach S 2013 Correlated X-ray scattering dataset, silver nanoparticles. Harv. Dataverse Netw. (10.7910/DVN/23244)

[17] Cohen AE Ellis PJ Miller MD Deacon AM Phizackerley RP 2002 Cryocrystallography papers. J. Appl. Crystallogr. 35, 720–726. (10.1107/S0021889802016709) 24899734 PMC4041710

[18] Levard C Reinsch BC Michel FM Oumahi C Lowry GV Brown GE Jr 2011 Sulfidation processes of PVP-coated silver nanoparticles in aqueous solution: impact on dissolution rate. Environ. Sci. Technol. 45, 5260–5266. (10.1021/es2007758) 21598969

[19] Henke BL Gullikson EM Davis JC 1993 X-Ray interactions: photoabsorption, scattering, transmission, and reflection at *E* = 50-30,000 eV, *Z* = 1–92. Atomic Data Nucl. Data Tables 54, 181–342. (10.1006/adnd.1993.1013)

[20] Schröder GF Levitt M Brunger AT 2010 Super-resolution biomolecular crystallography with low-resolution data. Nature 464, 1218–1222. (10.1038/nature08892) 20376006 PMC2859093

[21] Neutze R Wouts R van der Spoel D Weckert E Hajdu J 2000 Potential for biomolecular imaging with femtosecond X-ray pulses. Nature 406, 752–757. (10.1038/35021099) 10963603

[22] Spence JCH Weierstall U Chapman HN 2012 X-ray lasers for structural and dynamic biology. Rep. Prog. Phys. 75, 102601 (10.1088/0034-4885/75/10/102601) 22975810

